# The Landscape of the Emergence of Life

**DOI:** 10.3390/life6020020

**Published:** 2016-05-16

**Authors:** Sohan Jheeta

**Affiliations:** NoR HGT & LUCA, 1 Scott Hall Crescent, Leeds, LS7 3RB, UK; sohan7@ntlworld.com; Tel: +44-113-262-8767

Is it unrealistic to presuppose that all of the steps that could lead to the formation of life could occur in one setting? On the face of it, it would seem that life was made in a single setting but when one delves deeper into life’s origin, it appears to be multifaceted. So unravelling the physical and chemical mechanisms that led to the origin of life on Earth is still the same monumental task as when Oparin and Haldane put forward their hypothesis pertaining to the chemical origin of life in 1929.

Currently there are four front runners, namely the panspermia, vesicle, metabolism and genetic first hypotheses. Panspermia contends that life was primarily made elsewhere in the Universe and then was delivered, ready-made, on to the Earth; the hypothesis does not elaborate on how life was made in space and therefore falls outside the remit of this paper. In recent times the ‘vesicle first hypothesis’ has been gaining ground on the premise that some sort of a ‘bag’ was necessary in order to bring most of the necessary organic molecules into the vicinity of one another and cause them to react with each other. It has been put forward that such vesicles could either be made on the sea shores of early Earth or that long, up to 18C atoms, chains of hydrocarbons were delivered onto the Earth via meteorites, comets and asteroids. Also delivered would have been other “life friendly” chemicals, often termed biogenic molecules such as cyanate ions, OCN^-^; formyl radicals, HCO; methyl formate, H_3_COHCO; isocyanic acid, HNCO; formic acid, HCOOH; formamide, HCONH_2_; formaldehyde, H_2_CO; and methane, CH_4_, which would have been made in the vastness of space [1,2,3]. As of April 2016, ~190 molecules have been detected in both the interstellar medium and circumstellar envelope [4]. The metabolism first hypothesis promotes that simple biogenic monomers such as H_2_CO came first; this molecule is important during the formation of all important sugars (e.g., ribose). These monomers were made in the alkaline hydrothermal vents located on the sea floor (e.g., Lost City hydrothermal vent field in the mid-Atlantic Ocean). Proponents of this hypothesis have been able to simulate alkaline vents in the laboratory and have synthesised organic molecules, e.g., methanol (CH_3_OH) and formic, acetic (CH_3_COOH) and pyruvic (CH_3_COCOOH) acids from CO_2_ dissolved in sea water, eventually leading to a more complex chemistry that gave rise to the origin of life [5]. Finally, the genetic first hypothesis upholds that an information system in the form of RNA came first, followed by proteins and then DNA. This hypothesis for the origin of life can be broadly divided into two phases; namely the preRNA and RNA chemistry worlds, although in reality no such distinct division exists; it is probably more akin to a continuum of reactions occurring individually, leading to pathways, to cycles and hypercyles with both positive and negative feedbacks and then self-generating some of the necessary relevant molecules. As the latter hypothesis forms the major area of interest for the Network of Researchers on Horizontal Gene transfer and the Last Universal Common Ancestor (NoR HGT & LUCA), it will be the focus of this submission.

The division of preRNA and RNA in the genetic first hypothesis is introduced for two reasons. Initially, there appears to be a distinct ‘step-over’ line between the two chemistries. Further, there are many uncertainties surrounding preRNA chemistry, meaning what were the precursors which led to formation of nucleotides, in particular nitrogenous bases, namely adenine (A), cytosine (C), guanine (G) and uracil (U)? It is these nitrogenous bases in conjunction with ribose sugar (C_5_H_10_O_5_) and phosphate $(H_{3}P_{4}^{-}$) group which form the backbone of an RNA molecule. Some scientists posit that the RNA molecule was probably made from other precursor molecules, such as threose nucleic acid (TNA), peptide nucleic acid (PNA) or some other, as yet undiscovered, analogue molecules. Unlike RNA, any preRNA chemistry is not preserved in the form of chemical fossils; we can only make a best guess at the chemical precursors of preRNAs. Even if we succeed in identifying the preRNA chemistry by which the initial RNAs were made, it does not necessarily mean that they were synthesised via these “test tube” steps.

If we start with the premise that RNA can be made, then our understanding of the origin of life becomes marginally more comprehensible. This is because RNA exhibits two very important facets, in that it can undertake catalytic activity, meaning it acts as a ribozyme [6]; and it can carry a genetic code as testified by RNA viruses (e.g., flu virus) and, coding (e.g., mRNA) and non-coding (e.g., transfer and ribosomal RNAs) RNA related activities displayed in all cellular life forms on Earth. More importantly, the RNA molecule is conserved in all cellular life forms, especially in ribosomes and this means that genetic histories of these life forms can be traced back beyond the point when the three domains of life (Archaea, Bacteria and Eukarya) first emerged around 3.5 × 10^9^ years ago [7], as evidenced by both carbon and sulphur isotope (^13^C and ^34^S respectively) fractionation studies. In addition, it is extrapolated that the said three domains evolved from a hypothetical organism called the Last Universal Common Ancestor (LUCA) which existed ~3.8 × 10^9^ years ago.

LUCA is a theoretical construct. It was an entity which was, for all intents and purposes, believed to be ‘alive’ and possessing a DNA-based repository genetic code; a DNA replication system; and was able to carry out aminoacylation, transacylation and peptide synthesis—*i.e*., it possessed a functional ribosome of some kind [8]. In effect, LUCA was a DNA-based organism [9] emerging from what is often referred to as an ‘RNA world’ [10] which probably dates from 4.0 to 3.8 x 10^9^ years. During the RNA world there would have been a ‘preLUCA world’ where essentially quasi-entities encased in clay bubbles flourished; such entities would have had simple RNA genomes, replication apparatus (largely involving tRNA) and probably even rudimentary peptides [11]. Some scientists have posited that, as preLUCAs were encased in these clay bubbles, they would have been held in the vicinity of one another thus forming immobile communities, which allowed them to exchange genes horizontally, leading to the emergence of LUCAs [12]. The preLUCAs would have been exceptionally slow and hugely sluggish because RNA was an inefficient repository molecule being unstable and prone to misinterpretation during transcription and translation; similarly, they were ineffective catalysts due to the low levels of reactions they could carry out.

Eventually with the advent of a cell membrane, preLUCAs would have left their clay bubble domains and become the free floating entities, LUCAs. During the reign of LUCAs horizontal gene transfers (HGT) were rife and were aided by strong atmospheric lightning which was a frequent feature of the very early Earth from 4.0 to 3.8 × 10^9^ years ago [13]. During such events, LUCA membranes became highly permeable due to ‘aqueous holes’ traversing the lipid bilayer of the membranes in strong electric fields brought about by the lightning [14]. Naturally this led to the exchange of genetic material between different LUCAs. In the process some LUCAs would have received detrimental genes and thus would have been ‘weeded’ out and their content recycled; conversely those receiving ‘healthy’ genes would evolve further—is this the period when Darwinian evolution began? Empirical studies on nucleic acids seem to suggest this may be so. The continued evolution of LUCAs led to the development of the more stable DNA molecule as the custodian of genetic codes. Simultaneously the proteins which were also present during the reign of LUCAs quickly evolved from being rudimentary ones, with the result that they became superfast and super-efficient as far as catalytic activities were concerned; the vast majority, if not most, of the ribozymatic activities in cellular life forms being discontinued and thus consigned to history. Ultimately LUCAs became transformed into the three domains of life we recognise today. Evolutionarily, both preLUCA and LUCA lost out to the more efficient DNA/protein organism, leaving them to their own fate; in terms of rock fossils all traces of preLUCA and LUCA disappeared. The exceptions were RNA chemical relics which acted as a subservient shadow of DNA in the cells of all living entities on Earth—a reminder that there once were LUCA organisms.

At the same time as LUCAs, viruses also containing RNA evolved during a parallel evolution. In addition, genetic material was passed from viruses to LUCA and probably *vice* *versa* via transduction processes (which would also include “membrane vesicle transfer” and “genetic transfer” agents) and, in fact, there is evidence to suggest that the replication apparatus in all life forms on Earth is of viral origin [15]. Further elaboration on viruses is curtailed due to time and space.

The text in this paper forms a backdrop to the landscape of the emergence of life and outlines some of the areas for consideration and discussion of the NoR HGT & LUCA meeting being held on 3–4 November 2016 at the Open University, Milton Keynes, UK.
